# A practice framework to enhance the implementation of the Policy on Integration of Mental Health Care into primary health care in KwaZulu-Natal province

**DOI:** 10.4102/phcfm.v11i1.1865

**Published:** 2019-04-10

**Authors:** Esther N. Hlongwa, Maureen N. Sibiya

**Affiliations:** 1 KwaZulu-Natal College of Nursing, Pietermaritzburg, South Africa; 2 Faculty of Health Sciences, Durban University of Technology, Durban, South Africa

## Abstract

**Background:**

Mental health care at primary health care (PHC) still remains a challenge despite the Policy on Integration of Mental Health Care into PHC which was developed in 1997 at the time when the White Paper for the Transformation of the Health System in South Africa was published. The White Paper made provision for a new health care system based on the principles of the PHC approach to care. This was followed by the promulgation of the *Mental Health Care Act* No. 17 of 2002 which is based on the principle that mental health care should be integrated into PHC; however, there have been challenges with regard to the implementation of this policy.

**Aim:**

This study aimed to analyse the implementation of Policy on Integration of Mental Health Care into PHC with the ultimate aim of developing a practice framework for PHC nurses to enhance such implementation in KwaZulu-Natal (KZN).

**Setting:**

The study took place in selected health districts in KZN, namely, Ugu, eThekwini, iLembe and uMgungundlovu.

**Methods:**

A qualitative approach using grounded theory design was used to develop a practice framework to enhance the implementation of Policy on Integration of Mental Health Care into PHC. A theoretical sampling method was used to select the sample from PHC managers, operational managers and professional nurses for the collection of data. The sample consisted of 42 participants. Data were collected by means of one-on-one interviews and focus group interviews. Strauss and Corbin’s approach of data analysis was used for analysing data. The paradigm model was used as a guide to develop a practice framework to enhance the implementation of the Policy on Integration of Mental Health Care into PHC in KZN.

**Results:**

This study found that integration of mental health care into PHC is understood as a provision of comprehensive care to mental health care users using either a supermarket approach or a one-stop-shop approach at PHC clinics. Strategies that are used at PHC clinics in KZN ensure that the integration of mental health care into PHC is implemented, includes the screening of all patients that come to the PHC clinic for mental illness, fast tracking of mental health care users once they have been assessed, and found to be mentally ill and management of all mental health care users as patients with chronic diseases.

**Conclusion:**

The practice framework developed identifies comprehensive mental health care being offered to mental health care users using either a supermarket approach or a one-stop-shop approach, depending on the availability of staff with a qualification in psychiatric nursing science.

**Keywords:**

Primary health care; mental health care; health-related policies; integration of care; comprehensive care.

## Introduction

The Policy on Integration of Mental Health Care was promulgated in 1997 with the White Paper for the Transformation of health services. According to the Policy on Integration of Mental Health Care into primary health care (PHC), mental health care users should receive treatment at the clinic, near to where they live and mental health care services must be integrated into general care at PHC.^1^ The *Mental Health Care Act* No. 17 of 2002 was also promulgated in South Africa and is based on a principle that mental health care should be integrated into PHC and should take place near to where people live.^2^ PHC re-engineering in South Africa is a national strategy which was launched in 2010 by the National Minister of Health, which is aimed at improving PHC by promoting health care which is more practical, integrated and based on the needs of the population.^3^ In a PHC clinic, nurses are responsible for the management, administration and counselling of patients.^3^ This demands that the professional nurses working at the clinic should be able to assess all patients, identify patients with mental illness and provide the necessary treatment.

In an attempt to implement the policy, the National Department of Health released guidelines for the management of mental disorders in 2006.^4^ In response to National Department of Health guidelines, KwaZulu-Natal (KZN) Department of Health was the first province to release treatment protocols and an essential drug list for the management of mental disorders which was based on the guidelines issued by the Department of National Health in 2007.^5^ In 2013, the National Mental Health Policy Framework and Strategic Plan 2013–2020 were developed for South Africa to guide the implementation of policy framework.^6^ Since the publication of the Policy on Integration of Mental Health Care into PHC, there have been challenges that have been reported.^7,8,9^ This includes insufficient support and information to guide PHC nurses on the implementation of the policy and the lack of skills and time to provide quality care.^10,11,12,13^ These challenges are affecting the implementation of the Policy on Integration of Mental Health Care into PHC; hence, the study aimed to analyse the implementation of policy with the ultimate aim of developing a practice framework for PHC nurses to improve such implementation in KZN.

## Research methodology and design

### Study design

The study adopted a qualitative approach using grounded theory design.

### Study setting

KwaZulu-Natal province has 11 districts. The study took place in four health districts in KZN province, namely, uGu, uMgungundlovu, eThekwini and iLembe. eThekwini district is urban and incorporates the largest city in KZN with a total population of 3.5 million. uMgungundlovu district is located in Pietermaritzburg and is largely urban with a total population of 1 052 730. uGu district is mostly rural with a total population of 733 228. iLembe district is mostly rural and has population of 630 464.

### Sampling strategy

A three-stage sampling was applied as follows:

*The 1st stage*: It was selecting health districts in KZN to be used in the study. Four health districts were purposely selected according to geographic location and included central (eThekwini), midlands (uMgungundlovu), north (iLembe) and south (uGu). eThekwini and uMgungundlovu health districts are situated in urban areas and iLembe and uGu health districts are situated in rural areas. All four districts had implemented the Policy on the Integration of Mental Health Care into PHC at their clinics.

*The 2nd stage*: It was to select clinics located within districts of eThekwini, uGu, uMgungundlovu and iLembe. The researcher purposefully selected one central and accessible clinic per district that provides comprehensive care and had implemented the Policy on Integration of Mental Health Care. The clinics that were purposefully selected were KwaDabeka Clinic at eThekwini district, Gamalakhe Clinic at uGu district, Mbalenhle Clinic at uMgungundlovu district and Ndwedwe Clinic at iLembe district.

*The 3rd stage*: It was selecting participants who were going to take part in the study. The non-probability sampling approach that was used in this research was purposeful sampling and theoretical sampling because PHC managers, operational managers and professional nurses are best suited to answer the research question. The process of purposeful sampling used in this study involved the researcher selecting nurse managers, operational managers and professional nurses who are involved in the management of mental health care users at PHC clinics. Theoretical sampling means participants were selected on the basis of their ability to contribute to the development of a practice framework.^14^

### Data collection

After the researcher obtained ethical clearance, data were collected from four PHC clinics in four districts. The sample consisted of 42 participants of whom four were PHC managers, six operational managers and 32 professional nurses. One-on-one interviews were conducted with 10 participants, four PHC managers and six operational managers. Focus group discussions were conducted with the professional nurses, who at least had 2 years’ experience in a PHC setting. These professional nurses were directly involved in implementing the policy at the operational level. A set of open-ended questions were used as a guide to facilitate the one-on-one interviews and focus group discussions. Data obtained during the one-on-one interviews and focus group discussions were voice recorded using field notes as a backup. Data collection continued until saturation of data was reached. Data collection and analysis occurred concurrently and took over 3 months. This allowed the researcher to constantly compare similarities and variations to determine common categories and subcategories and go back to interview participants to fill in gaps identified during analysis. The interviews and focus group discussions were transcribed within 12 hours of being conducted.

### Data analysis

The researcher used Strauss and Corbin’s approach to data analysis. According to Strauss and Corbin,^15^ analysing data using grounded theory is a process which reduces raw information into concepts that can be coded and designated as subcategories using three coding procedures: open coding, axial coding and selective coding.^16^ Open coding was achieved by breaking down, examining, comparing, labelling and categorising data. During open coding, questions were asked and data were examined until the stage that no new categories emerged from the process. Axial coding was used to connect categories of data of similar characteristics found when using open coding.^15^ Categories which shared similar characteristics were merged into the categories found in Strauss and Corbin’s paradigm model.^17^ Selective coding was used to integrate categories of data that have been gathered to form a model.^15^ Selective coding is the process of integrating and refining categories. It involves the identification of a core category from which the practice framework will be developed. The paradigm model, as described by Strauss and Corbin, is an organising tool that connects subcategories of data to the central idea so as to help the researcher to think systematically about the data, and allows the researcher to ask questions on how categories of data relate to each other. Paradigm model includes phenomenon, causal conditions, context, action, interactional strategies and consequences.^15^ All major categories were integrated to form a larger practice framework.

### Trustworthiness

To ensure trustworthiness of the study, the researcher ensured that the research met the criteria described by Strauss and Corbin’s grounded theory.^15^ It should, firstly, be valid, reliable and credible; secondly, the research process should be adequate; and thirdly, research findings should be empirically grounded. Three coding procedures were used, namely, open coding, axial coding and selective coding. Open coding was accomplished by breaking down data, examining, and comparing, labelling and categorising data to develop concepts. Axial coding was used to connect categories according to their characteristics and similarities and emerging them to the categories found in Strauss and Corbin’s paradigm model. Selecting coding was used to integrate and refine categories. To ensure richness of data, triangulation was implemented by using multiple methods of collecting data which included one-on-one interviews and focus group interviews, memos and field notes. Credibility was also achieved through multiple reviews of the voice recordings and field notes during data analysis.

## Ethical consideration

Ethical approval for conducting this study was obtained from the ethics committee (Ethical clearance reference number: REC 46/16). Permission to conduct the study was requested from the District Manager and the KZN Department of Health. All participants in the study were given a letter of information and consent form to consent to participate in the study. Their participation was voluntary and they were told that they had a right to withdraw from the study at any time without any penalty. The researcher requested permission to voice record the interviews. The participants were informed that they would not receive monetary benefits for participating in the study. Extra precautions were taken to safeguard the participants with regard to anonymity and confidentiality. Their names were not mentioned and recorded during discussions. They were advised of the confidentiality and anonymity of the discussion and responses.

## Results

Integration of mental health care into PHC was seen in this study as comprehensive care which can be given to mental health care users using either a supermarket approach or one-stop-shop approach depending on the availability of nurses with psychiatric nursing qualification. These approaches to care are seen as strategies used to implement integration of mental health care into PHC. The practice framework also describes the enabling environment that will allow integration of mental health care to take place effectively.

Strategies used for integration of Mental Health Care into PHC are described below:

### Supermarket approach

Some participants viewed integration of mental health care into PHC as being a supermarket approach. This implies the provision of all services that the mental health care user needs by different nurses or health care providers in one clinic. This means that the mental health care user moves from one consulting room to another for each condition needing to be treated. This is supported by the following excerpts:

‘Mental health care services are provided by one nurse who has been trained. If during assessment I discover that the patient has mental illness, then I refer them to the psychiatric nurse in another consulting room. I know it’s time consuming but the patient gets all services in one place.’ (Participant 2, female, nurse manager)

‘The patient gets all the services that she needs in one clinic, only has one trip to the clinic and get all the services in one day but from different nurses.’ (Participant 4, female, professional nurse)

### One-stop-shop approach

Most participants viewed integration of mental health care as a one-stop-shop approach to care; mental health care users are managed holistically looking at physical, social and psychological aspects of care by one nurse. If the mental health care user comes to the clinic, the nurse also checks for other diseases such as tuberculosis and diabetes. This is what the participants said: ‘Every patient that enters the PHC level must be assessed for mental illness by one nurse so that she can be treated or referred*’* (Participant 4, female, nurse manager).

‘When a patient comes you see and treat them and you don’t refer them. If you as a nurse encounter mentally ill patient, you need to attend that patient and not just refer them.’ (Participant 21, female, professional nurse)

‘All the mental health care users should be treated the same as other patients when they enter the clinic and there should not be days scheduled specifically for mental health patients. Fair and equal treatment to all patients.’ (Participant7, female, operational manager)

**FIGURE 1:**
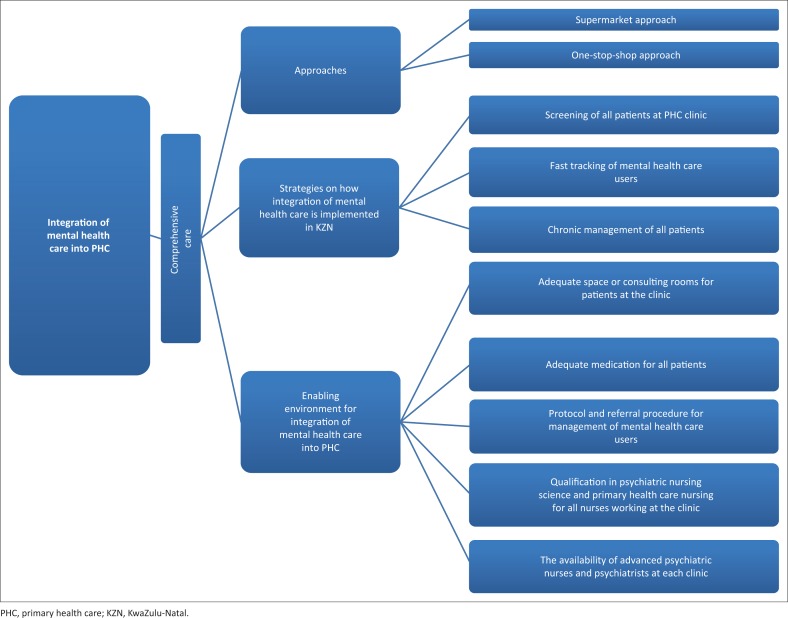
The practice framework for integration of mental health care into primary health care.

### The strategies for integration of mental health care into primary health care at KwaZulu-Natal

The practice framework describes three strategies that can be used at PHC clinics in KZN to ensure that integration of mental health care into PHC is implemented: (1) screening of all patients for mental illness, (2) fast tracking of mental health care users and (3) chronic management of all patients including mental health care users.

### Screening all patients for mental illness

Clinics where a one-stop-shop approach is used indicated that all patients that come to the clinic are assessed for mental illness and if found to be mentally ill are managed by a professional nurse or referred if it is beyond the scope of that nurse. They expressed their views by stating that:

‘In this clinic all patients are screened for all the illnesses if they are found to be mentally or physically ill they are treated by all professional nurses. Although I don’t have the qualification in mental health but I do screen all the patients. We screen all patients in this clinic.’ (Participant 10, female, operational manager)

*‘*We screen all patients in this clinic for mental illness and treat all patients. We have a screening tool called PC101 which allows us to screen all patients’ (Participant 4, female, nurse manager).

### Fast tracking of mental health users

All participants indicated that at the clinic there is a fast-tracking system in place. When mental health care users come to the clinic, screening is done and if the client is found to be mentally ill, they are re-routed to the mental health practitioner who will provide further treatment or doctor who will prescribe medication. If the mental health care user is not stabilised, they are referred to the next level of care which is for 72-h assessment period, which is available at the district hospital.

According to Sokhela, Makhanya, Sibiya and Nokes^18^, the fast queue strategy was introduced in South Africa to resolve challenges in a range of health care services, including PHC services, to reduce long waiting periods at the clinics. This includes treatment and follows up of patients and their families with mental illness, referral of patients for drug abuse, sexual abuse, child abuse and other crisis situations. This was expressed in the following excerpts:

‘When mental health users come to this clinic for chronic medication, they are fast tracked, we don’t want them to wait for a long time, we fast track them so that they don’t get angry. These patients get angry when they wait for a long time.’ (Participant 42, female, professional nurse)

#### Chronic management of patients at the clinic

The results of this study revealed that all PHC clinics which participated in the study had processes in place that allowed mental health care users, who are on chronic medication, to be referred to a professional nurse who does integrated management of chronic illnesses which includes management of mental illness. This is consistent with the provisions of the model called ‘symptom based integrated approach’ at PHC clinics (PC101) which was commissioned and published by the National Department of Health which provides guidelines for the management of all chronic illnesses, including management of mental health care users who have chronic mental illness. The main aim of PC101 is to standardise the approach to adult patients who are presenting with chronic symptoms or those who came for review of their chronic medication.^19^ Participants said:

‘Mental health care users are treated like any chronic patient at the clinic, the only thing we do for them, is to fast track them because they don’t want to sit in the queue for a long time, they get agitated.’ (Participant 5, female, operational manager)

‘Patient is seen holistically, if they have hypertension and mental illness; they get all the treatment from the same professional nurse.’ (Participant 19, female, professional nurse).

‘In this clinic, all patients with chronic illness are treated the same. They are all checked and treated by a PHC nurse who does chronic patients. This is very good because nurses don’t only see one kind of patients; they get to see variety of all patients.’ (Participant 5, female, operational nurse)

### The enabling environment that will allow integration of mental health care into primary health care to take place effectively

The framework identifies the following factors as an enabling environment that may result in successful implementation of the Policy on Integration of Mental Health Care into PHC.

#### Adequate space or consulting rooms for patients at the clinic

Adequate space and consulting rooms for counselling and interviewing clients were viewed as an important condition that needs to be in place before integration of mental health care can take place. Participants cited that enough space and consulting rooms provide adequate privacy and allow for confidentiality of information between mental health care users and health workers. This is what the participants said:

‘Having adequate rooms for mental health care users to be counselled and consulted is very important to maintain privacy and confidentiality. Privacy should be ensured for patients coming to the clinic.’ (Participant 20, female, professional nurse)

‘Environment should be conducive and safe for counselling and management of patients’ space must be able to accommodate difficult patients and allows for management of agitated and aggressive patients in the clinic.’ (Participant 21, female, professional nurse)

*‘*We need more consulting rooms and bigger space especially when other members of the multidisciplinary teams visit the clinic; we really need more space to accommodate everybody.’ (Participant 7, female, Operational nurse manager).

#### Adequate medication for all patients

Adequate medication supplies for all patients were viewed as an important condition that needs to be in place before integration of mental health care can take place. The participants stated that enough medication supplies allow PHC nurses to give treatment to mental health care users and prevent relapse and re-admission to mental institutions. Participants said:

‘There should be enough treatment available for all patients at the clinic. Enough medication needs to be stored in every consulting room even for night duty or weekends.’ (Participant 1, female, nurse manager).

‘Shortage of medication and at times some of the drugs used by mental health care users is not in stock. Medication is important for managing mental health care users all the time.’ (Participant 21, female, professional nurse)

*‘*Shortage of medication and at times some of the drugs used by mental health care users is not in stock.’ (Participant 8, female, operational nurse manager).

‘Having enough medication to cater for all mental health care users. At the moment we do not have enough and type of medication to cater for mental illness like the medication in institutions which are dedicated for mental health care.’ (Participant 25, female, professional nurse)

#### Protocol and referral procedure for the management of mental health care users

Protocols and referral procedures for the management of mental health care users were viewed as an important condition that needs to be in place before integration of mental health care can take place. The participants stated that they feel there must be step-by-step procedures put in place by management for mental health care users and that this must be communicated to all staff at the clinic. They further recommended that there should be engagement of community members and other stakeholders in the community including police on how to manage mental health care users so that they support the policy. This was expressed in the excerpts in the following excerpts:

‘There should be step-by-step protocols on how to manage and refer mental health care users in each clinic, and there must be enough resources to implement the protocol.*’* (Participant 20, female, Professional nurse).

‘If the mental health care user cannot be treated, then the mental health care user needs to be referred and there must be a proper protocol in place to determine when the patient must be referred.’ (Participant 8, female, operational nurse manager)

*‘*There should be protocols that are communicated throughout the department by management*’* (Participant 24, female, professional nurse).

#### Qualification in psychiatric nursing science and Primary Health Care nursing science for all nurses at the clinic

Qualification in psychiatric nursing science and PHC for all nurses working at the PHC clinic was viewed by all participants as an important condition that needs to take place before integration of mental health care into PHC can be achieved. Participants cited that each and every nurse at PHC clinics should have the necessary skills to be able to manage mental health care users. This was noted in the following quotes:

‘Every professional nurse at the clinic should have knowledge of mental illness and must be able to recognise signs of mental illness.’ (Participant 1, female, nurse manager).

‘PHC nurses must be trained in both Psychiatric Nursing Science and PHC to be able to understand and manage all patient including mental health care users.’ (Participant 7, female, operational nurse manager).

*‘*It’s important to train nurses in psychiatric nursing, so that they don’t misdiagnose mental illness.’ (Participant 1, female, nurse manager).

### The availability of advanced psychiatric nurses and psychiatrists

Participants stated that the availability of psychiatrists and advanced psychiatric nurses to provide support and mentoring of PHC staff who do not have qualification in psychiatric nursing would provide an enabling environment for effective integration of mental health care into PHC. All clinics cited shortage of staff especially psychiatric nurses, specialist psychiatrist and advanced psychiatric nurses as the main factor hindering the implementation of integration of mental health care into PHC. In some clinics, a psychiatrist only comes once a week and in some clinics a general practitioner sees all patients including mental health care users. The following are the participants’ words: ‘I think that PHC nurses need support and mentoring from the psychiatrists or advanced psychiatric nurses. At the moment there is only one PHC nurse with advanced psychiatry’ (Participant 7, female, operational nurse manager). ‘The psychiatrist only comes once a week and this not enough. I think we are having problems to implement this policy because of lack of support*’* (Participant 33, female, professional nurse). *‘*I think PHC nurses need specialist support for effective integration of mental health care into PHC*’* (Participant 9, female, operational nurse manager).

## Discussion

In KZN, the Policy on Integration of Mental Health Care into PHC has been implemented despite the challenges that have been reported. Integration of mental health care into PHC was reported as provision of comprehensive care which is being offered to mental health care users using either a supermarket approach or a one-stop-shop approach at PHC clinics depending on whether the PHC nurses have qualification in psychiatric nursing science or not.

The study revealed that in clinics where a supermarket approach is used, mental health care users are screened and then referred to a professional nurse who has a psychiatric nursing science qualification and who only manages mental health care users at the clinic. The participants expressed concern that this it might cause problems to have one professional being the only person who manages mental health care users, and felt that this approach leads to those professional nurses being overloaded with work and also being labelled as mentally ill themselves because of the nature of the work they do. This is supported by Mkhize and Kometsi^20^ who argue that despite integration of mental health care into PHC, there are concerns that non-psychiatric nurses sometimes opt to leave the task of attending to mental health care users to one person, who sometimes then feel stigmatised themselves by their colleagues because of the nature of their work.

The findings of this study are consistent with the findings of the study that was conducted by Vhuromu and Davhana-Maselesele^21^ which revealed that the supermarket approach of rendering health services involves treating patients as they arrive without grouping them to come to the clinic on a particular day or time and further argue that one type of health education which is to be given to almost all patients is repeated many times and this can be time-consuming and tiresome to PHC nurses.

A one-stop-shop approach to care, where mental health care users are managed holistically by one nurse, was reported to take place in clinics where their PHC nurses had a qualification in psychiatric nursing. The results of this study are consistent with the findings of the study that was conducted by Sibiya and Gwele,^22^ who found that IPHC was viewed by participants as the provision of services by one nurse and that the patient receives comprehensive care in one clinic. In South Africa, PHC comprehensive package indicates that clinics should provide comprehensive IPHC service through a one-stop-shop approach for a minimum number of hours.^23^ In support of one-stop-shop, French et al.^24^ state that this approach to care is the provision of all services at one site which allows for clinic efficiency, is convenient for the patient and allows patients to receive effective health care services. On the contrary, a one-stop-shop approach may result in staff being overloaded with work.^23^ Sibiya and Gwele^22^ argue that one can never be sure if a one-stop-shop approach saves time or not and further state that it may take longer for one nurse to provide all the services.

Strategies that are used at the PHC clinic to ensure that integration of mental health care into PHC is implemented in KZN province include screening of all patients that come to the PHC clinic for mental illness, fast tracking of mental health care users once they have been assessed and found mentally ill, and chronic management of all patients, including mental health care users, using the PC101 model. Dube and Uys^7^ warn that lack of knowledge and skills among PHC nurses are factors that contribute to poor management of mental health care users and further argue that shortage of PHC nurses without a psychiatric nursing science qualification lead to poor quality mental health care. Petersen et al^10^ state that despite the release of guidelines, there are gaps in the management of mental health care users at PHC clinics, especially in rural areas, namely, insufficient support for PHC nurses which could lead to poor identification of mental disorders at PHC clinics. Inadequate skills, poor support and training of PHC nurses are major factors undermining successful integration of mental health care into PHC.^9,20,25,26^

The framework developed in this study identifies an enabling environment that may result in successful implementation of the Policy on Integration of Mental Health Care into PHC to include adequate space or consulting rooms for patients at the clinic, adequate medication for the treatment of mental health care users, protocols and referral procedures for the management of mental health care users, qualification in psychiatric nursing science and PHC nursing science for all nurses at the clinic and the availability of advanced psychiatric nurses and psychiatrists at each clinic to support PHC nurses. Despite the challenges pertaining to PHC nurses’ ability and skills to implement the Policy on Integration of Mental Health Care into PHC, it should be remembered that PHC nurses should engage in lifelong learning through the use of workshops, conferences and future studies. Although the policy is implemented in KZN, PHC nurses feel that there are conditions that need to be in place for the implementation of the Policy on Integration of Mental Health Care into PHC to be effective.

## Conclusion

This study attempted to analyse the implementation of the Policy on Integration of Mental Health Care into PHC with the ultimate aim of developing a practice framework that will enhance the implementation of the policy in KZN, South Africa. The practice framework identifies approaches used to provide mental health care, the strategies that are currently used and the enabling environment for the implementation of the policy. The developed practice framework could assist PHC nurses in improving the quality of mental health care at PHC clinics in KZN, South Africa. PHC nurses should be encouraged to further their knowledge, skills and competence throughout their professional lives.

### Recommendations

It is recommended that the KZN Department of Health should develop protocols, guidelines and communication strategies on how to implement the policy. Managers need to be capacitated with needed skills to provide leadership in this matter. Lack of supervision by specialists can also hamper the provision of quality mental health care at PHC level. Therefore, it is recommended that at least there must be a psychiatrist and advanced psychiatric nurses who will supervise and mentor PHC nurses in providing quality mental health care. Lack of resources, space, equipment, lack of political will and increased workload were identified as major factors hindering the implementation of the policy. Therefore, it is recommended that PHC facilities should receive an increased budget because of additional services that are now offered at PHC clinics. There is urgent need to relook at the PHC curriculum so that new PHC graduates will understand integration of mental health care into PHC, their roles and how to function in such an environment.

## References

[1] Republic of South Africa White Paper for the transformation of the health system in South Africa. Pretoria: Government Printer; 1997.

[2] National Department of Health (of South Africa) Mental Health Care Act, No.17. Government Gazette Pretoria: Government Printers; 2002.

[3] National Department of Health (of South Africa) Re-engineering primary health care in South Africa: Discussion document. Government Gazette Pretoria: Government Printers; 2010.

[4] National Department of Health (of South Africa) National guidelines for the management of mental health care user in South Africa. Pretoria: Government Printers; 2006.

[5] KwaZulu-Natal Department of Health Standard treatment protocols for common mental health conditions. Pietermaritzburg: Government Printers; 2007.

[6] National Department of Health (of South Africa) National Mental Health Policy Framework and Strategic Plan 2013–2020. Pretoria: Government Printers; 2013.

[7] Dube FN, Uys LR Primary health care nurse’s management practices of common mental health conditions in KwaZulu-Natal, South Africa. Curationis. 2015;38(1):1–10. 10.4102/curationis.v38i1.1168 PMC6091611 26244460

[8] Kigozi FN, Ssebunnya J Integration of mental health into primary health care in Uganda: Opportunities and challenges. Mental Health Fam Med. 2009;6:37–42. PMC2777598 22477886

[9] Burns JK Implementation of the Mental Health Care Act 2002 at district hospitals in South Africa: Translating principles into practice. S Afr Med J. 2008;98(1):46–49. 18270641

[10] Petersen I, Joshua Sebunnya J, Bhana A, Baillie K, Mha PP Research Programme Consortium. Lessons from case studies of integrating mental health into primary health care in South Africa and Uganda. Int J Mental Health Syst [serial online]. 2011 [cited 2017 Mar];5(8). Available from: http://www.ijmhs.com/content/5/1/8 10.1186/1752-4458-5-8 PMC3096573 21496242

[11] Awenza AD, Read UM, Otori-Attah A, Doku VCK, Akpalu B, Osei A From mental health policy development in Ghana to Implementation: What are the barriers? Afr J Psychiatry. 2012;13:184–191. 20957317

[12] Draper CE, Lund C, Kleintjies S, Funk M, Omar M, Flisher AJ Mental health policy in South Africa: Development, process and content. Health Policy Plan. 2009;24(5):342–356. 10.1093/heapol/czp027 19561012

[13] Williams DR, Herman A, Stein DJ, et al Twelve-month mental disorders in South Africa: Prevalence, service and demographic correlate as in the population-based South African and health study. Psychol Med. 2008;38(2):211–220. 10.1017/S0033291707001420 17903333 PMC2718686

[14] Creswell JW Qualitative inquiry and research design: Choosing among the approaches. 3rd ed. Thousand Oaks, CA: Sage; 2012.

[15] Strauss A, Corbin J aBasics of qualitative research: Grounded theory, procedures and techniques Newbury Park, CA: Sage; 1990.

[16] Chenitz WC, Swanson JM From practice to grounded theory: Qualitative research in nursing Menlo-Park: Addison-Wesley; 1986.

[17] Strauss A, Corbin J Basics of qualitative research: Techniques and procedures for developing a grounded theory. Thousand Oaks, CA: Sage; 1998.

[18] Sokhela DG, Makhanya NJ, Sibiya MN, Nokes KM Experiences of fast queue health care users in primary health care facilities in eThekwini district, South Africa. Curationis. 2013;36(1):1–8. 10.4102/curationis.v36i1.60 23902165

[19] National Department of Health (of South Africa) Primary Health Care 101 (PC101): Symptom based integrated approach to adult primary health care. Pretoria: Government Printers; 2016.

[20] Mkhize N, Kometsi MJ Community access to mental health services: Lessons and recommendations. S Afr Health Rev. 2008; 2008(1):103–113.

[21] Vhuromu EN, Davhana-Maselesele M Experiences of primary health care nurses in implementing in implementing integrated management of childhood illness strategy at selected clinics of Limpopo Province. Curationis. 2009;32(3):60–71. 10.4102/curationis.v32i3.1224 20225745

[22] Sibiya MN, Gwele NS An analysis of the meaning of integrated primary health care from the KwaZulu-Natal primary health care context. Curationis. 2009;32(2):31–37. 10.4102/curationis.v32i2.924

[23] National Department of Health (of South Africa) The comprehensive Primary Health Care Service Package for South Africa. Pretoria: Government Printers; 2001.

[24] French S, Cope CM, Graham A, Gerressu M, Salisbury C, Stephenson JM One stop versus collaborative integration: What is the best way of delivering sexual health services? Sex Transm Infect. 2006;82:202–206. 10.1136/sti.2005.018093 16731668 PMC2564738

[25] Van Wijk E, Traut A, Julie H Environmental and nursing staff factors contributing to aggressive and violent behaviour of patients in mental health facilities. Curationis. 2014;37(1):1–9. 10.4102/curationis.v37i1.1122 25686162

[26] Awases MH, Bezuidenhout MC Roos JH Factors affecting the performance of professional nurses in Namibia. Curationis. 2013;36(1):1–8. 10.4102/curationis.v36i1.108 23718720

